# Linkage of routinely-collected healthcare data and bespoke research questionnaire data to best serve NHS patient study participants.

**DOI:** 10.23889/ijpds.v7i3.1893

**Published:** 2022-08-25

**Authors:** Joanna McLaughlin, Andrew Judge

**Affiliations:** 1 University of Bristol

## Objectives

Pre-surgical ‘health optimisation’ programmes aim to improve patients’ health and to reduce their need for surgery. Routinely-collected data does not provide sufficient insight into whether or how these programmes meet their intended outcomes. The aim was to link routinely-collected NHS data with bespoke research questionnaire data to address this insufficiency.

## Approach

A study was designed to identify and collect data from a patient cohort eligible for a health optimisation programme (offering weight management and smoking cessation support) to determine programme uptake and patient outcomes. Patient and public involvement (PPI) group engagement revealed support for the use of data linkage between routinely-collected NHS data and study questionnaires, and shaped the study proposal. The HDRUK-supported regional ‘Systemwide Dataset’ of NHS routinely-collected data would provide demographics, healthcare usage and clinical outcomes. Questionnaires would provide data on patient decision-making and engagement with weight management or smoking cessation and detailed pain, function, wellbeing and activation measures.

## Results

The study received Health Research Authority approval and recruited six Participant Identification Centres. PPI co-production of the study’s participant-facing documents addressed the need for appropriate description of the novel data linkage process for potential participants. The data linkage design will offer an important advantage of a reduction in the participants’ time and recall accuracy needed in completing questionnaires due to the ability to omit duplicate demographic and healthcare usage fields. Further potential advantages include the ability to compare participants with patients who decline participation in the questionnaires, and long-term follow up of patient outcomes due to the continuation of routinely-collected data. Challenges have been faced in operationalizing the data collection and linkage processes due to decisions over data controllership of the combined dataset.

## Conclusion

Linkage between routinely-collected NHS data and bespoke research questionnaires was deemed ethically acceptable, and could offer significant advantages in combining demographics, healthcare usage, outcomes and experience data of a cohort. Systemic decision-making to specify and streamline acceptable processes for this data linkage are important next steps.

